# A New Sign in the Diagnosis of Intra-Cranial Affections

**Published:** 1893-03

**Authors:** 


A NEW SIGN IN THE DIAGNOSIS OF INTRA-CRANIAL AFFECTIONS.—In the January number of the *Anales del Circulo Medico Argentin,* Dr. Masi has an interesting article describing a new diagnostic symptom. The great importance of thoracic vibrations—their absence or exaggeration is appreciated in detecting diseases of the thoracic cavity. The same vibrations which occur during articulation are conveyed to the base of the skull, and thence to the vertex, where they can be detected as readily as over the thorax. Apply the palm of the hand to the head and one to the thorax, and the vocal fremitus will be as distinct to the one hand as to the other. If this sign, through its variations, can be utilized to help in the diagnosis of intra-thoracic affections, cannot it be likewise utilized in the diagnosis of intra-cranial affections ? This is the question which the author tried to solve. A patient was brought to the hospital, who, through injury, sustained a fracture at the base of the skull with ramifications as far as the upper part of the temporal regions on both sides. All the common signs of fracture of the base, such as bleeding and escape of arachnoid fluid from the ears and nose, etc., were present. Over the left temporal region there was found to be depression of about two centimeters in diameter. The patient rallied, and on the eighth day, as the doctor was examining the head, the patient spoke and a distinct fremitus was perceived over the whole head, except over the depressed bone in the left temporal region. This was noticed by members of the hospital staff and regarded as a strange phenomenon, which would, perhaps, enable one to apply it to other brain and skull lesions. In a normal head, therefore, the vibrations should be distinctly perceived over all portions of the skull, but in those cases where there is increased pressure, such as tumors, abscesses, and hemorrhages, or where there is thickening of the dura or adhesions between the brain and the skull through the dura, or fractures, absence of this vibration would be expected. The author describes the sign as Cranial Vibrations, and awaits further corroboration of his discovery.—*Buffalo Medical and Surgical Journal.*

